# Effects of GABAa receptor antagonists on motor behavior in pharmacological Parkinson's disease model in mice

**DOI:** 10.14814/phy2.13081

**Published:** 2017-03-29

**Authors:** Karla De Michelis Mograbi, Ana Carolini Ferreira de Castro, Jainny Aniely Rocha de Oliveira, Patrick Jean Barbosa Sales, Luciene Covolan, Eliane Aparecida Del Bel, Albert Schiaveto de Souza

**Affiliations:** ^1^ Laboratory of BiophysiopharmacologyUniversidade Federal de Mato Grosso do SulCampo GrandeBrazil; ^2^ Laboratory of NeurophysiologyUniversidade Federal de São PauloSao PauloBrazil; ^3^ Laboratory of PhysiologyUniversidade de São PauloSao PauloBrazil

**Keywords:** Bicuculline, catalepsy, flumazenil, GABA

## Abstract

The aim of this study was to evaluate the effects of two gamma‐amino butyric acid (GABA)a receptor antagonists on motor behavioral tasks in a pharmacological model of Parkinson disease (PD) in rodents. Ninety‐six Swiss mice received intraperitoneal injection of Haloperidol (1 mg/kg) to block dopaminergic receptors. GABAa receptors antagonists Bicuculline (1 and 5 mg/kg) and Flumazenil (3 and 6 mg/kg) were used for the assessment of the interaction among these neurotransmitters, in this PD model. The motor behavior of the animals was evaluated in the catalepsy test (30, 60, and 90 min after drugs application), through open field test (after 60 min) and trough functional gait assessment (after 60 min). Both Bicuculline and Flumazenil were able to partially reverse catalepsy induced by Haloperidol. In the open field test, Haloperidol reduced the number of horizontal and vertical exploration of the animals, which was not reversed trough application of GABAa antagonists. Furthermore, the functional gait assessment was not sensitive enough to detect motor changes in this animal model of PD. There is an interaction between dopamine and GABA in the basal ganglia and the blocking GABAa receptors may have therapeutic potential in the treatment of PD.

## Introduction

Caused by loss of dopaminergic neurons in substantia nigra, Parkinson's disease (PD) leads to a very well described disruption of extrapyramidal motor functions. Therefore, dopaminergic replacement is the main treatment in PD to relieve the neurotransmission symptoms on the alteration in nigrostriatal pathway (Jankovic 2002).

The PD pharmacological treatment is currently performed with dopamine agonists and Levodopa (L‐dopa). These drugs are quite effective in the early stages of the disease, treating neuromotor symptoms without altering the disease progression. However, it still causes several adverse effects such as nausea, vomiting, hypotension, and motor complications in the long term (Jenner 2003). Therefore, it is necessary to develop new pharmacological approaches that are able to act in all stages of the disease, diminishing the risk of appearance of involuntary movements (Jenner 2003).

The major inhibitory neurotransmitter in the central nervous system (CNS) is the gamma‐amino butyric acid (GABA). Inhibition is mediated by the ionotropic and metabotropic receptors, located pre or postsynaptically (Owens and Krigstein 2002). One of the best‐known ionotropic receptors within the circuitry of the basal ganglia is the GABAa receptor. Its inhibitory effect is through increasing chloride conductance (Cl^−^) (Iacono et al. 2013). These receptors consist of a combination of eight subunits: *α* (1–6) *β* (1–3), *γ* (1–3), *δ*,* ε*,* θ*,* π*, and *ρ* (1–3) (Jacob et al. 2012; Johnston 2013). The most prevalent composition in humans is *α*1 *β*2 *γ*2, where GABA binds between *α*1 and *β*2 (Bergmann et al. 2013).

Chen and Yung (2004) reported that the decrease in dopamine released from the substantia nigra pars compacta increases striatopallidal GABAergic activity that, in turn, reduces the spontaneous firing of the globus pallidus and modifies its pattern, contributing to the PD hypokinetic symptoms. Thus, inhibition of this pathway through the GABAa receptor, located in the globus pallidus, could alleviate these symptoms, expressing evidence of a direct relationship between the GABAa receptor and PD.

No study so far has correlated blocking of GABAa receptors with signs and PD motor symptoms. In this work, we set out to test the effect of two of GABAa receptor antagonists – Bicuculline and Flumazenil, given intraperitoneally, in catalepsy induced by Haloperidol. In addition, we studied the possible changes in motor behavior arising from the interaction of these compounds, through the open field test, and gait disturbance, through functional gait test.

## Materials and Methods

### Animals

The protocols used herein were approved by the Ethics Committee on the use of animals/CEUA from the Universidade Federal do Mato Grosso do Sul (UFMS, protocol # 586/2014).

Ninety‐six adult male Swiss mice, obtained from UFMS Breeding Colony, weighing between 30 and 40 g, were used in this study. They were housed in groups of four animals/plastic cage in the colony room that was maintained on a 12‐h on/12‐h off light cycle, with lights on at 0600 am. All experiments were conducted in the light phase. Mice received water and food ad libitum. The room temperature was kept constant (23 ± 1°C).

### Pharmacological tests

A single dose of the dopamine receptor antagonist, Haloperidol (Janssen‐Cilag, São Paulo, Brazil) was used: 1 mg/kg; for GABAa receptor antagonists two doses of Bicuculline (Sigma, São Paulo, Brazil) were used: 1 and 5 mg/kg; and two of Flumazenil (Cristália, São Paulo, Brazil): 3 and 6 mg/kg. The drugs were dissolved in saline 0.9% and injected intraperitoneally, Haloperidol and Bicuculline, were applied at a volume of 10 mL/kg, and Flumazenil was administered at 30 mL/kg. The interval between each drug administration was 30 min. The doses used were based on previous studies (Mierzejewski et al. 2013; Rodriguez‐Landa et al. 2013).

The experimental design consists of two groups: in the experiment I, the GABAa receptor antagonist used was Bicuculline and in the experiment II, Flumazenil. Each of these two experiments consisted of six groups (*n* = 8 mice/each). Group 1 (control) received two Saline injections. Group 2 received Saline followed by the GABAa receptor antagonist (the lowest dose). Group 3 received Saline followed by the GABAa receptor antagonist (the highest dose). Group 4 received Haloperidol followed by the Saline. Group 5 received Haloperidol followed by the GABAa receptor antagonist (the lowest dose). Group 6 received Haloperidol followed by the GABAa receptor antagonist (the highest dose). The experimental group design is shown in Table 1 for experiment I and Table 2 for experiment II.

**Table 1:** Experimental group design Experiment I

Groups	Haloperidol (1 mg/kg)	Bicuculline (1 mg/kg)	Bicuculline (3 mg/kg)
Saline + Saline	0	0	0
Sal + Bic low	0	1	0
Sal + Bic high	0	0	1
Hal + Saline	1	0	0
Hal + Bic low	1	1	0
Hal + Bic high	1	0	1

Sal, saline; Bic low, Bicuculline lowest dose; Bic high, Bicuculline highest dose; Hal, Haloperidol. 0 – not given, 1 – given.

**Table 2:** Experimental group design Experiment II

Groups	Haloperidol (1 mg/kg)	Flumazenil (3 mg/kg)	Flumazenil (6 mg/kg)
Saline + Saline	0	0	0
Sal + Flu low	0	1	0
Sal + Flu high	0	0	1
Hal + Saline	1	0	0
Hal + Flu low	1	1	0
Hal + Fluc high	1	0	1

Sal, saline; Flu low, Flumazenil lowest dose; Flu high, Flumazenil highest dose; Hal, Haloperidol. 0 – not given, 1 – given.

### Functional assessment

#### Catalepsy test

The bar method was used to assess catalepsy. The bar was made with horizontal glass (diameter 0.5 cm) and elevated 4.5 cm from de floor. The mouse was placed with both forelimbs on the bar and the time it stayed in this position was recorded (seconds). The catalepsy time ended when the animal's forelimbs touched the ground or the mouse climbed up on the bar. The test's result is the average time spent in three attempts (maximum 300 sec). The test was conducted 30, 60, and 90 min after the second drug (or Saline for the Control group) administration.

#### Open field test

The open field test was conducted 60 min after the second drug administration, in a cylindrical arena of 40 cm in diameter with translucent acrylic walls of 30 cm height, placed on a wooden base covered by white Formica, which has been subdivided into 12 quadrants of 104.7 cm^2^ each. In our study, we evaluated the following parameters: frequency of the behavior of horizontal exploration, which is the number of quadrants crossed in 5 min, and vertical exploration, which is the number of rearing in the same 5‐min period.

### Functional gait assessment

For this analysis, the forelimbs and hind limbs were painted and the footprints printed when the mouse walked on a wooden platform (5 cm width and 29 cm length), covered with an ink‐absorbent paper, toward a little dark house located at end of the platform. In order to characterize the direction of locomotion, the footprints of the forelimbs were used.

Three measurements were taken from the footprints records: (1) the stride length (in centimeters) was determined by the average distance between the central region of two consecutive footprints; (2) the anteroposterior and lateral‐lateral deviation between the ipsilateral footprints of the front and rear limbs; and (3) the base of support was determined by the distance between footprints from both hind limbs. The functional gait assessment was carried out just 60 min after the second drug application.

### Statistical analysis

The catalepsy test was analyzed with the nonparametric Friedman test followed by Student–Newman–Keuls post hoc test. The comparison between groups, regarding the variables, catalepsy time, horizontal exploration, vertical exploration, step length, support base, lateral‐lateral and anteroposterior deviations was performed using Kruskal–Wallis test, followed by the post hoc test Student–Newman–Keuls. Statistical analysis was performed using the statistical package SigmaPlot version 12.5 (Systat Software Inc., São Paulo, Brazil), considering a significance level of the 5%.

## Results

### Experiment I

#### Catalepsy

**Figure 1:**
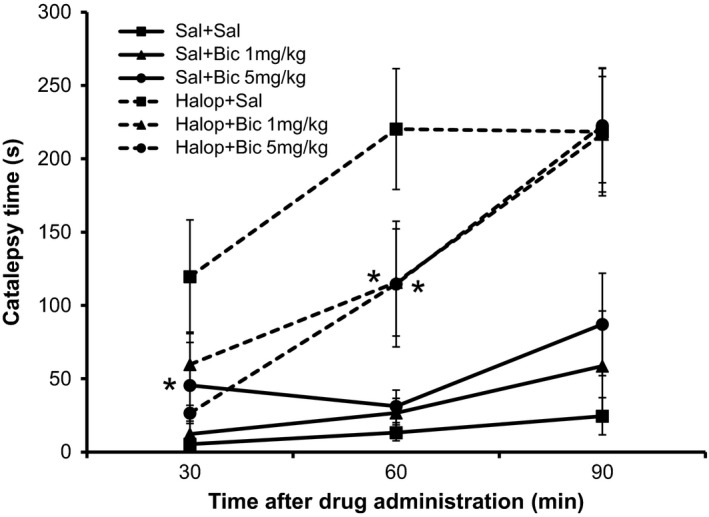
Graphic related to catalepsy time in each experimental group, at each time of the catalepsy test, in Experiment I. *Significant difference from Haloperidol + Saline group (Student–Newman–Keuls post hoc test, *P* < 0.05).

The results for the catalepsy time in different groups and moments of analysis, in Experiment I are shown in Figure 1.

##### Comparison between moments

Catalepsy time at times 60 and 90 min after drugs application was significantly higher than that at 30 min in Saline + Saline and Haloperidol + Bicuculline 5 mg/kg groups (*P* = 0.018, both). Furthermore, in the Saline + Bicuculline group (5 mg/kg) despite of the *P*‐value being 0.038, the difference was not found when it was performed the Student–Newman–Keuls post hoc test.

##### Comparison between groups

There was a difference among experimental groups, at all catalepsy times examined (*P* < 0.001).

In the three moments, the animals in groups that received Haloperidol showed a higher catalepsy time than those who received Saline as first drug. However, after 30 min the animals from group Haloperidol + Bicuculline 5 mg/kg showed a significant reversal of catalepsy when compared with Haloperidol + Saline group, but partial, compared with Saline + Saline group.

After 60 min, the animals in Haloperidol + Bicuculline groups 1 and 5 mg/kg showed a significant reversal of catalepsy as compared with Haloperidol + Saline group, though partial, compared to the Saline + Saline group. Moreover, after 90 min there was no significant reversal of catalepsy time.

#### Open field

**Figure 2:**
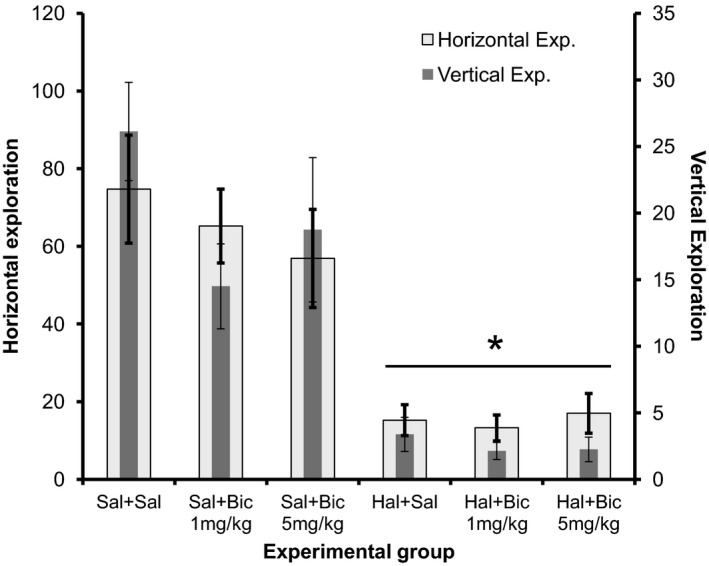
Graphic related to horizontal and vertical exploration at open field test in each experimental group – Experiment I. *Significant difference from groups that receive saline as the first drug (Student–Newman–Keuls, *P* < 0.05).

The results related to the open field test in different groups, in Experiment I, are illustrated in Figure 2.

The difference between groups was significant for both horizontal and vertical explorations, considering that the groups where Haloperidol was used as the first drug the animals explored less than the ones which received first Saline (*P* < 0.001). Bicuculline was unable to reverse the motor effects of Haloperidol.

#### Functional gait assessment

The results of the evaluation of gait in different groups, in Experiment I are shown in Table 3.

**Table 3:** Results related to functional gait assessment, at each experimental group Experiment I

Groups	Functional gait assessment			
	Base of support	Stride length	LatLat D	AntPost D
Saline + Saline	2.70 ± 0.10a	2.84 ± 0.18a	0.66 ± 0.19a	0.89 ± 0.15a
Sal + Bic 1 mg/kg	2.61 ± 0.21a	3.35 ± 0.18a	0.52 ± 0.05a	0.81 ± 0.11a
Sal + Bic 5 mg/kg	2.54 ± 0.11a	3.14 ± 0.17a	0.49 ± 0.04a	0.67 ± 0.06a
Hal + Saline	2.59 ± 0.12a	2.81 ± 0.12a	0.53 ± 0.04a	0.75 ± 0.14a
Hal + Bic 1 mg/kg	2.28 ± 0.21a	2.59 ± 0.17a	0.47 ± 0.02a	1.04 ± 0,07a
Hal + Bic 5 mg/kg	2.19 ± 0.27a	2.60 ± 0.25a	0.59 ± 0.07a	0.93 ± 0.18a
*P* value	0.467	0.031	0.828	0.324

Sal, saline; Bic, Bicuculline; Hal, Haloperidol. Results are presented as mean ± standard error. Data are measured in centimeters. “a” indicate not significant differences between the experimental groups (posttest Student–Newman–Keuls test, *P* > 0.05).

There were no significant difference between experimental groups for any variable studied: base of support (*P* = 0.467), step length (*P* = 0.031), lateral‐lateral (*P* = 0.828), and anteroposterior deviation (*P* = 0.324).

### Experiment II

#### Catalepsy

**Figure 3:**
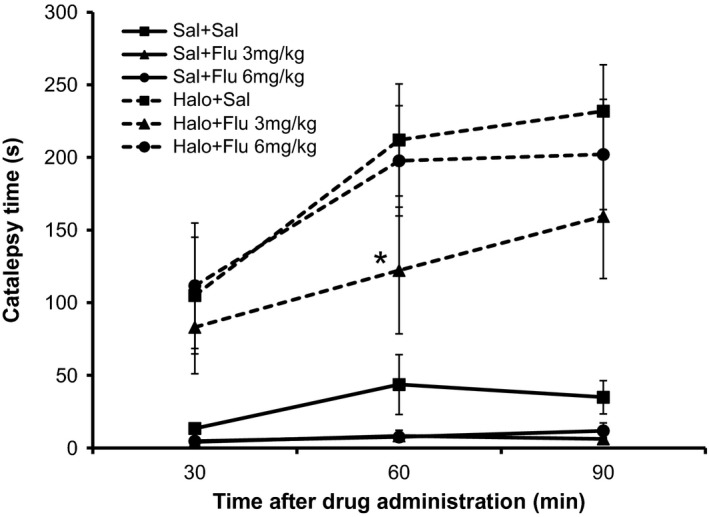
Graphic related to catalepsy time in each experimental group, at each time of the catalepsy test, in Experiment II. *Significant difference from Haloperidol + Saline group (Student–Newman–Keuls post hoc test, *P* < 0.05).

The results for the catalepsy time in different groups and moments of analysis, in Experiment II are shown in Figure 3.

##### Comparison between moments

Catalepsy time in the moments 60 and 90 min after the drug administration was significantly higher than that at 30 min in the Saline + Saline and Haloperidol + Saline (*P* = 0.030, and 0.005, respectively). On the other hand, there was no difference between the analyzed moments to Saline + Flumazenil (3 and 6 mg/kg) and Haloperidol + Flumazenil 3 and 6 mg/kg groups.

##### Comparison between groups

There was difference between the experimental groups, in all catalepsy moments analyzed.

At all times, the animals that received Haloperidol showed a higher catalepsy time than those who received Saline as first drug (*P* < 0.001). However, only at 60 min the animals of group Haloperidol + Flumazenil 3 mg/kg really showed a significant reversal of catalepsy, compared with Haloperidol + Saline group (*P* < 0.001), but partial compared with Saline + Saline group. At 30 and 90 min, there was no significant reversal of catalepsy time.

#### Open field

**Figure 4:**
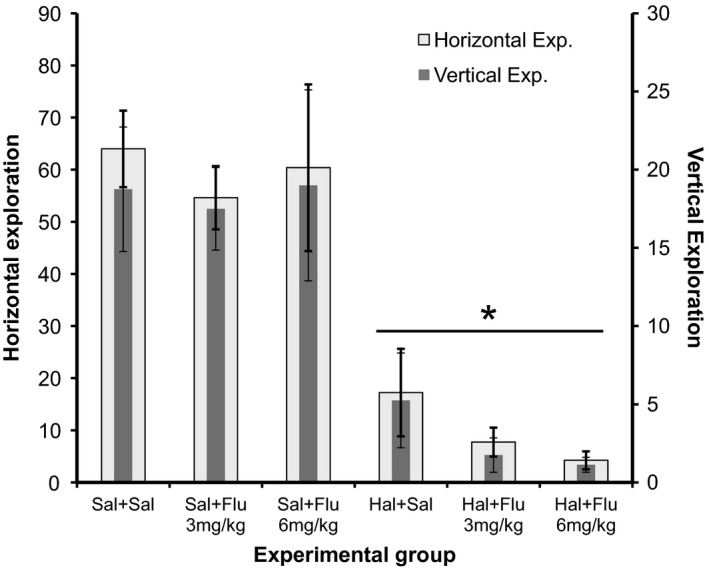
Graphic related to horizontal and vertical exploration in open field test at each experimental group – Experiment II. *Significant difference from groups that receive saline as the first drug (Student–Newman–Keuls, *P* < 0.05).

The results related to the open field test in different groups, in Experiment II are shown in Figure 4.

The difference between the groups was significant for both horizontal and vertical exploration, and the groups receiving Haloperidol as the first drug the animals explored less than those which first received Saline (*P* < 0.001). Flumazenil was unable to reverse the motor effects of Haloperidol.

#### Functional gait assessment

The results concerning the functional gait assessment in the different groups, in Experiment II are shown in Table 4.

**Table 4:** Results related to functional gait assessment, at each experimental group Experiment II

Groups	Functional gait assessment			
	Base of support	Stride length	LatLat D	AntPost D
Saline + Saline	2.66 ± 0.14a	3.29 ± 0.22a	0.50 ± 0.07a	0.46 ± 0.07f
Sal + Flu 3 mg/kg	2.60 ± 0.15a	3.04 ± 0.13a	0.46 ± 0.07a	0.87 ± 0.14c
Sal + Flu 6 mg/kg	2.43 ± 0.10a	3.37 ± 0.12a	0.51 ± 0.12a	0.52 ± 0.05e
Hal + Saline	2.41 ± 0.09a	2.14 ± 0.32c	0.45 ± 0.04a	0.66 ± 0.06d
Hal + Flu 3 mg/kg	2.64 ± 0.09a	2.12 ± 0.18c	0.46 ± 0.06a	1.25 ± 0.13a
Hal + Flu 6 mg/kg	2.65 ± 0.09a	2.74 ± 0.22b	0.51 ± 0.03a	0.94 ± 0.06b
*P* value	0.361	<0.001	0.880	<0.001

Sal, saline; Flu, Flumazenil; Hal, Haloperidol. Results are presented as mean ± standard error. Data are measured in centimeters. Different lowercase letters in the columns indicate significant differences between the experimental groups (posttest Student–Newman–Keuls test, *P* < 0.05).

There was a significant difference for the variables step length (*P* < 0.001) and anteroposterior deviation (*P* < 0.001), since the animals that received Haloperidol as the first drug had the lowest stride length than those who received Saline, but the animals who received the highest dose of Flumazenil (6 mg/kg) showed a partial recovery of this length. All groups in anteroposterior deviation were different.

## Discussion

In this study, in Experiment I, the application of Haloperidol causes catalepsy since it significantly enhanced the time mice spent on the bar compared to saline controls. Bicuculline at 1 and 5 mg/kg dose produced a partial reversal at 30 and 60 min after administration. In the open field test, administration of Haloperidol significantly decreased both the horizontal and the vertical exploration of the animals; however, Bicuculline was not able to reverse these motor effects. Finally, in Experiment I, Haloperidol did not result in significant motor changes in the functional gait assessment.

In Experiment II, only at 60 min after Flumazenil application at a dose of 3 mg/kg the catalepsy caused by Haloperidol was partially reversed. In the open field test, Flumazenil was also unable to reverse the motor effects of Haloperidol. Unlike the observation in Experiment I, Haloperidol led to a decrease in the step length of the animals, which was partially reversed by Flumazenil at a dose of 6 mg/kg. In relation to the anteroposterior deviation, the results were inconclusive.

Studies have shown a strong relationship between the GABAergic neurotransmission and PD (Xue et al. 2010; Chen et al. 2013). For example, Xue et al. (2010) showed that microinjection of GABAa receptor antagonist (Gabazine) in the globus pallidus increased the firing rate of pallidal neurons, demonstrating a high level of GABAergic activity in globus pallidus in parkinsonian state. They reported that, probably after lesion with 6‐OHDA, many reasons may explain this excitatory effect in globus pallidus: (1) postsynaptic GABAa receptors expression increased or (2) enhanced GABA release toward globus pallidus, (3) less efficient GABA reuptake, (4) changes in intraneuronal Cl^−^ level, (5) excitatory inputs reduction or, (6) change in some peptide action. Then, they suggested that blocking GABAa receptors in globus pallidus increases its spontaneous firing rate (Xue et al. 2010).

Changes in the GABAa receptor system may cause motor dysfunction. Depletion of dopamine induces to overactivity of the striatopallidal pathway, which leads a decrease in activity of the globus pallidus neurons. And then, this reduction in globus pallidus activity contributes to excessive inhibition of basal ganglia targets regions (Chen and Yung 2004). This hypoactivity of the globus pallidus is characterized by a decrease in the spontaneous firing rate, in which it is difficult to find a pallidal neuron with a spontaneous firing (Chen et al. 2013). This modification suggests that it is the central origin of parkinsonian symptoms (Chen and Yung 2004).

In this study, we proposed to assess whether the blockade of GABAa receptors in the globus pallidus would reverse the typical motor disorders of PD in an animal model, using two specific antagonists: Bicuculline and Flumazenil. These antagonists have been widely used in studies aimed at understanding the PD pathophysiology (Ondo and Hunter 2003; Ondo and Silay 2006; Parga et al. 2007; Turner et al. 2008), and the relationship of the GABAa receptor with other diseases such as anxiety, memory alterations and stress (Whissell et al. 2013; Martin et al. 2014; Palotai et al. 2014).

Catalepsy is the main dysfunction found in animal models of PD. Thus, when we applied the GABAa receptor antagonist, Bicuculline, we observed the reversal of catalepsy time partially because there was no return to the level of the control animals (Saline + Saline). The drug used in the animal model, Haloperidol, is a potent neuroleptic drug, which causes extrapyramidal symptoms, similar to PD and this also occurs when used in humans (Nayebi and Sheidaei 2010).

The reversal not only occurred partially, but it was also brief, that is, this occurred only during the first two moments of evaluation – 30 and 60 min after the application. This result corroborates the study by Tostes et al. (2013) who observed that the peak action of Bicuculline is ~15 min after administration. This indicates that in this study the action of bicuculline was already declining since the first test was only done 30 min after administration.

Petri et al. (2013) demonstrated that activation of GABAa receptors in the subthalamic nucleus relieves motor symptoms caused by damage to dopaminergic neurons in the substantia nigra pars compacta. Since Bicuculline and Flumazenil were applied intraperitoneally, leading to a systemic action involving GABAa receptors in various CNS structures, their administration, besides blocking the globus pallidus, may have acted on the subthalamic nucleus, keeping it hyperactive, not fully reversing catalepsy as seen in our results, although its GABAergic inputs were reduced (Kalia et al. 2013).

Although it is already known that the GABAa receptor subunits play different roles and have different pharmacology (Johnston 2013) and distribution on the basal ganglia (Pirker et al. 2000), their relationship is still obscure. Therefore, this might be another factor that probably influences the results in this study. Thereby, knowledge of this relationship in order to select the most suitable drug is also necessary (Brichta et al. 2013).

Some studies have used Bicuculline to test the interaction of other drugs that cause catalepsy, with the GABAergic system. For instance, in a study that investigated new treatment strategies for psychiatric disorders and sought to explain the action of 5‐HT receptor, the Bicuculline was able to reverse catalepsy, even when applied intraperitoneally (Desphande et al. 2001) that was unlike from our results.

In experiment II, Flumazenil partially reversed the catalepsy 60 min after administration. This is also a fast‐acting drug, with a half‐life around 0.7–1.3 h, with its effect lasting up to 5 h (Votey et al. 1991). In a study that aimed to verify the catalepsy characteristics caused by a hypnotic drug non‐benzodiazepine – Zolpidem, animals pretreated with Flumazenil (intraperitoneal) had their catalepsy time significantly lower than those who received only the Zolpidem (Mierzejewski et al. 2013).

The open field test is a simple test to evaluate the global motor activity, willingness to explore, and anxiety behaviors (Peña et al. 2013) and has been used in studies designed to assess the improvement of motor performance in animal models of PD by using various types of drugs (Peña et al. 2013; Miao et al. 2014).

In motor evaluation through the open field test, neither of the two antagonists used in the study were able to reverse the decline of both horizontal and vertical explorations, caused by Haloperidol. This corroborates the findings of the Rodriguez‐Landa et al. (2013) study, showing that neither Bicuculline nor Flumazenil was able to improve the motor activity impaired by the human amniotic fluid and a mixture of eight fatty acids (Rodriguez‐Landa et al. 2013).

Studies have shown that Flumazenil can improve motor activity altered by drugs that interact with the GABAergic system, assessed by the open field test. In a study aimed to evaluate the mechanism of action of the active principle Tilianin isolated from an active methanol extract of *Agastache mexicana*, it was used the open field test and Flumazenil, to prove its action on GABAa receptors, where the antagonist was able to block the change in motor activity (González‐Trujano et al. 2015). Another study, aimed to assess the Phytol mechanism of action, also used the Flumazenil and the open field test, and evaluated its interaction with the GABAergic system through the improvement of motor activity after its administration (Costa et al. 2014).

In this study, during the functional gait analysis, Haloperidol was only able to cause motor perceptible change in Experiment II, which were partially reversed by Flumazenil, demonstrating that this motor assessment may not be sufficiently sensitive to detect the motor deficits in animal models of PD. This data agree with the study of Bonito‐Oliva et al. (2014) that evaluated the dynamics of animal gait with injury by 6‐OHDA, and found no significant difference in stride length, base of support, and lateral‐lateral and anteroposterior deviations when compared with control animals. However, they really demonstrated abnormalities in the step cycle, such as increase in swing phase and decrease in support phase, in both hind limbs, not tested in this study. In contrast, another study evaluated motor alterations presented by an animal model genetically modified – A53T mice, that showed a decreased stride length, corresponding to gait abnormalities found in patients with PD (Paumier et al. 2013).

New studies are needed to clarify the remaining issues, such as GABAa receptor blockade exclusively in the globus pallidus and the most appropriate time for the realization of the motor tests, in order to cover the peak action of these antagonists. Nevertheless, Haloperidol caused catalepsy and motor alterations in the animals, Bicuculline partially reversed the catalepsy at times 30 and 60 min, but it was not able to reverse the behavior alterations in the open field test, Flumazenil also partially reversed catalepsy at time 30 min and it was not capable to revert behavior alterations in the open field test. Therefore, GABAergic neurotransmission, in particular for GABAa receptors seems to be intrinsically involved in the circuitry of the brain's basal ganglia and its modulation may have important therapeutic potential in the treatment of PD.

## Conflict of Interest

None declared.
